# Influence of parkinson’s disease on complications and revisions in total hip and knee arthroplasty: insights from a matched pair analysis

**DOI:** 10.1007/s00264-024-06398-9

**Published:** 2025-01-24

**Authors:** Dominik Emanuel Holzapfel, Tobias Kappenschneider, Marie Farina Schuster, Stefano Pagano, Fady Azar, Sabrina Holzapfel, Matthias Meyer

**Affiliations:** 1 Department of Orthopaedic Surgery, Regensburg University Medical Center, Bad Abbach, Germany; 2 Department of Neonatology, University Children´S Hospital Regensburg, Hospital St. Hedwig of the Order of St John, University of Regensburg, Regensburg, Germany; 3 Department of Orthopaedic Surgery and Traumatology, Altmühltal Nature Park Clinics, Eichstätt, Germany

**Keywords:** Parkinson’s disease, TJA, TKA, THA, Postoperative complications, Revision surgery

## Abstract

**Purpose:**

The outcome of elective total joint arthroplasty (TJA) in patients with Parkinson’s disease (PD) is controversial due to the concomitant risk profile. This study investigated postoperative complications and revision rates following total hip (THA) and knee arthroplasty (TKA) in patients with PD.

**Methods:**

Ninety-six patients with PD undergoing THA or TKA were matched 1:1 with non-PD patients using propensity score matching for age, sex and comorbidity (Charlson Comorbidity index, CCI). Rates of revisions, medical and surgical complications were compared. Univariate and multivariate regression analyses were calculated.

**Results:**

PD patients exhibited higher rates of revision-surgeries within 90 days (13.5% vs. 5.2%; *p* = 0.048), medical complications (68.8% vs. 43.8%; *p* < 0.001) and surgical complications (40.6% vs. 21.9%; *p* = 0.005). Multivariate regression analysis confirmed PD as a significant risk factor for complications and long-term revision-surgeries.

**Conclusion:**

PD increases the risk of adverse outcomes following THA and TKA. Improvements in pre-operative planning and post-operative care are critical to the improvement of outcomes in this vulnerable population.

## Introduction

Parkinson's disease (PD) is a prevalent neurodegenerative disease with a progressive clinical course [1]. The age-dependent prevalence ranges from 1 to 5% [2–4], with advanced age being the primary risk factor [5]. According to the Global Burden of Disease Study, the age-standardized prevalence of PD has risen worldwide between 1990 and 2016 [6], with predictions suggesting a forthcoming ‘PD pandemic’ [7]. In parallel, the demand for TJA is increasing, as the ageing population contributes to a growing number of patients with osteoarthritis of large joints such as the hip and knee. [7–11]. Consequently, orthopaedic surgeons will encounter a higher number of PD patients requiring TJA.

Current literature identifies PD as a significant risk factor for perioperative morbidity in surgical patients [12, 13]. In context of elective TJA, PD has been associated with elevated complication rates, higher revision surgery frequencies and worse clinical outcomes [13, 14]. Neuromuscular dysfunctions such as resting tremor, bradykinesia, rigidity, shuffling gait, poor overall coordination and frequent falls—affecting up to 60% of PD patients annually, with 70% experiencing recurrent falls—may contribute to these outcomes. Corresponding issues, like concomitant medical conditions or osteoporosis, further complicate surgical outcomes [15–18]. Therefore, PD has been historically seen as a relative contraindication for TJA. In contrast, some studies report satisfactory functional outcomes with a life-changing improvement in quality of life and pain relief in PD patients following TJA [19, 20]. These findings highlight a lack of consensus regarding the safety and efficacy of TJA in this patient population. Despite increasing research, the specific impact of PD on postoperative complications, revision rates, and long-term outcomes remains underexplored.

This study aims to address the gap in the literature by systematically analyzing the impact of PD on postoperative outcomes following total hip (THA) and knee arthroplasty (TKA) in a matched patient cohort. The primary objective was to evaluate the risk of postoperative complications and revision surgery after TJA in patients with PD. Secondary objectives include examining the nature of complications.

## Materials and methods

### Study design and study population

This is a retrospective matched pair analysis based on a database derived from the hospital information system and the department’s joint registry. All patients who underwent primary elective THA or TKA between 2010 and 2023 were included, resulting in a consecutive series. Among these, 96 patients with PD were identified, using ICD-10 codes. Propensity score matching (1:1) was performed based on age, sex and comorbidity (Charlson Comorbidity index, CCI). The final dataset included 192 patients: 96 with PD and 96 non-PD controlls. Baseline characteristics were extracted from patient records.

### Endpoints and follow-up

The primary endpoints were reoperation within 90 days and postoperative complications. These adverse events (AEs) were categorized in surgical (periprosthetic joint infection, periprosthetic fracture, wound healing disorder and haematoma, aseptic loosening and subsidence, dislocation, instability and wear of mobile components, arthrofibrosis, range of motion limitation, anterior knee pain, tendon rupture and nerve injuries) and medical complications (cardiovascular, pulmonary, renal, thromboembolic, neurological and cerebrovascular AEs, delirium, urinary tract infection, electrolyte derangement and anemia requiring transfusion). Complications were defined and assessed using ICD-10 codes documented at discharge.

Secondary endpoint was a detailed breakdown of the most common causes of complications. In Addition, cases requiring transfer to intensive care units were recorded according to the Clavien-Dindo-Classification Grade IV [21]. This classification system ranks complications into five grades, based on the therapy used for correction. Grade IV complications are defined as life-threatening events requiring intensive care management [21]. Follow-up was conducted for 90 days postoperatively, with data extracted from hospital records, including subsequent complications and reoperations.

### Surgical techniques

All operations were performed in a single Department of Orthopedic Surgery of a University Medical Center. All patients received the same standardized treatment protocol for THA or TKA respectively, including a standardized rehabilitation programme. The majority of patients received spinal anaesthesia, with a smaller proportion receiving general or regional anaesthesia procedures. THA was conducted in the lateral decubitus position using a minimally invasive anterolateral approach, with cementless fixation. TKA was performed using a cemented technique through a medial parapatellar approach without patellar resurfacing.

### Data collection

Diagnoses coded at the time of hospitalization and discharge were extracted from the hospital information system (ORBIS®; Agfa Healthcare) including corresponding ICD-10-Codes. Diagnostic codes had been entered by professional clinical coders and were double-checked by physicians using information gathered from patients’ medical records. Patients with PD were identified by the ICD-10-Code G20. Complications were assessed according to the ICD-10-codes at the time of discharge. Hospital Frailty Risk Score (HFRS) and Charlson Comorbidity Index (CCI) were calculated according to the ICD-10-codes at the time of admission [22]. Further available data from our clinical information system were age, sex, operative procedure, length of stay, time of surgery, transfer to intensive care unit and reoperation.

### Statistics

Propensity score matching was performed to balance baseline characteristics between the PD and npn-PD groups- Data analysis was conducted using IBM SPSS Statistics (version 29.0.0), with a significance level set at p ≤ 0.05. Continuous variables are presented as means ± standard deviation (SD), as well as medians and interquartile ranges (IQR). Categorical data are reported as absolute (n) and percentage (%) frequencies. Continuous variables were tested for normal distribution (Shapiro–Wilk test), and since all variables were non-normally distributed, non-parametric methods, such as the Mann–Whitney U test, were applied for independent samples. Categorical variables were analyzed using the Chi-square test, and for small samples or low expected cell frequencies, Fisher’s exact test was applied. Univariate logistic regression was used to calculate odds ratios for relevant variables. Multivariate logistic regression models were used to assess tindependent effects on postoperative complications, including PD, type of arthroplasty (hip or knee), ASA score, HSRF, and BMI. This helped to screen confounders and to identify independent predictors of complications.

## Results

### Baseline characteristics

A total of 34455 patients were selected from the local hospital information system between January 2010 and Dezember 2023. From this cohort 14,505 cases were identified who had undergone elective THA or TKA surgery. Proportionally, 0.66% (96/14505) of these cases were affected by PD. After propensity score matching, the baseline characteristics of the PD and non-PD groups were balanced in terms of age, sex, and Charlson Comorbidity Index (CCI). Both groups showed a similar distribution for type of joint replacement (THA/TKA), body mass index (BMI), length of stay (LOS) in days, and time of surgery in minutes. The baseline characteristics are shown in Table 1.

**Table 1:** Baseline characteristics of the propensity score matched study cohorts

	**PD patients** (*n* = 96)	**non-PD patients** (*n* = 96)	**total population** (*n* = 192)	*p*-value
**♀** n (%)	49 (51.0)	55 (57.3)	104 (54.2)	0.385
**♂** n (%)	47 (49.0)	41 (42.7)	88 (45.8)	0.385
THA n (%)	38 (39.6)	39 (40.6)	77 (40.1)	0.883
TKA n (%)	58 (60.4)	57 (59.4)	115 (59.9)	0.883
Age (years)				
Mean (SD)	71.3 (8.4)	70.8 (8.9)	71.0 (8.7)	0.645
Median (IQR)	72.5 (10.0)	71.5 (13.0)	72.0 (12.0)	
BMI				
Mean (SD)	29.3 (4.9)	29.5 (5.4)	29.4 (5.1)	0.830
Median (IQR)	29.2 (7.3)	28.9 (7.0)	29.0 (7.1)	
ASA – Score				
Mean (SD)	2.7 (0.5)	2.3 (0.6)	2.5 (0.6)	** < 0.001**
Median (IQR)	3.0 (1.0)	2.0 (1.0)	3.0 (1.0)	
CCI				
Mean (SD)	3.2 (1.4)	3.0 (1.3)	3.1 (1.4)	0.381
Median (IQR)	3.0 (2.0)	3.0 (2.0)	3.0 (2.0)	
HFRS				
Mean (SD)	4.6 (3.7)	2.4 (2.0)	3.5 (3.2)	** < 0.001**
Median (IQR)	3.2 (4.6)	1.7 (2.1)	2.3 (2.8)	
LOS (days)	9.4 (3.5)	9.0 (4.3)	9.2 (4.0)	0.283
Mean (SD)	9.0 (3.0)	9.0 (2.0)	9.0 (3.0)	
Median (IQR)	*n* = 84	*n* = 96	*n* = 180	
time of surgery (minutes)	77.2 (24.1)	78.6 (22.6)	78.0 (23.3)	0.670
Mean (SD)	75.5 (35.5)	73.5 (26.0)	75.0 (28.5)	
Median (IQR)	*n* = 85	*n* = 96	*n* = 181	

*THA *Total hip arthroplasty, *TKA *Total knee arthroplasty, Age in years, BMI, *ASA *American Society of Anesthesiologists, *CCI *Charlson Comorbidity Index, *HFRS *Hospital Frailty Risk Score, *LOS *length of stay in days, time of surgery in minutes, bold values = *p* ≤ 0.05; LOS (*n* = 180) and time of surgery (*n* = 181) do not include the full data set of *n* = 192 cases, as the data from the in-house database were incomplete

### Primary outcome: revision rates

**Fig. 1:**
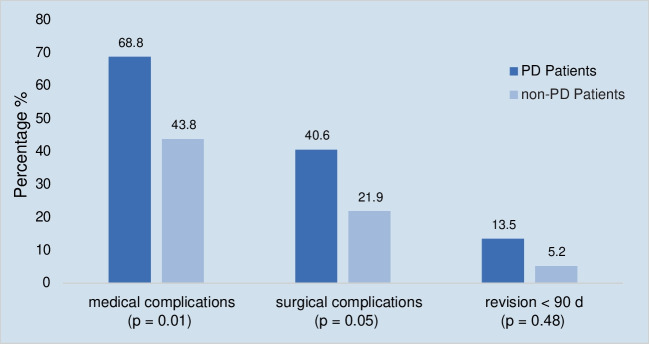
Medical complications, surgical complications and revision-rates in patients with Parkinson disease compared to control group. Medical complications include cardiovascular, pulmonary, renal, thromboembolic (TVT), neurological and cerebrovascular AEs, delirium, urinary tract infection, electrolyte derangement and anemia requiring transfusion, surgical complications include periprosthetic joint infection (PJI), periprosthetic fracture (PP#), wound healing disorder (WHD) and hematoma, aseptic loosening (AL) and subsidence, dislocation, instability and wear of mobile components, range of motion limitation (ROM↓) and anterior knee pain (AKP), tendon rupture and nerve injuries

PD patients following TJA had significantly higher revision-rates < 90 days [13.5% (13) vs. 5,2% (5); *p* = 0.048], compared to the control group (see Table 2 and Fig. 1).

**Table 2:** Postoperative complications after elective total joint arthroplasty of patients with or without underlying Parkinson’s disease

Total Joint Arthroplasty				
	**PD patients** *n* = 96 *n* (%)	**Non-PD patients** *n* = 96 *n* (%)	**total** *n* = 192 *n* (%)	*p*-value
revision surgeries (number of patients)				
total revision	27 (28.1)	7 (7.3)	34(17.7)	** < 0.001**
revision < 90 days	13 (13.5)	5 (5.2)	18 (9.4)	**0.048**
revision > 90 days	16 (16.7)	2 (2.1)	18 (9.4)	** < 0.001**
surgical complications (number of patients)	39 (40.6)	21 (21.9)	60 (31.3)	**0.005**
surgical complications (number of events)				
PP#	14 (14.6)	3 (3.1)	17 (8.9)	**0.005**
PJI	8 (8.3)	2 (2.1)	10 (5.2)	0.051
WHD/hematoma	10 (10.4)	3 (3.1)	13 (6.8)	**0.044**
AL/subsidence	10 (10.4)	0 (0.0)	10 (5.2)	**0.001**
AKP/ROM↓	7 (7.3)	8 (8.3)	15 (7.8)	0.788
dislocation (THA)	9 (9.4)	1 (1.0)	10 (5.2)	**0.009**
tendon rupture (TKA)	3 (3.1)	0 (0.0)	3 (1.6)	0.081
instability/wear	8 (8.3)	0 (0.0)	8 (4.2)	**0.007**
nerve injury	4 (4.2)	3 (3.1)	7 (3.6)	1.00
medical complications (number of patients)	66 (68.8)	42 (43.8)	108 (56.3)	** < 0.001**
medical complications (number of events)				
pulmonary AEs	10 (10.4)	1 (1.0)	11 (5.7)	**0.005**
decubitus	2 (2.1)	2 (2.1)	4 (2.1)	1.00
cardiovascular AEs	7 (7.3)	2 (2.1)	9 (4.7)	0.169
renal AEs	6 (6.3)	6 (6.3)	12 (6.3)	1.00
electrolyte derangement	28 (29.2)	11 (11.5)	39 (20.3)	**0.002**
anemia	43 (44.8)	29 (30.2)	72 (37.5)	**0.037**
delirium	14 (14.6)	4 (4.2)	18 (9.4)	**0.013**
urinary tract infection	4 (4.2)	2 (2.1)	6 (3.1)	0.683
TVT	4 (4.2)	0 (0.0)	4 (2.1)	0.121
other complications (number of events)				
Clavien-Dindo IV	2 (2.1)	3 (3.1)	5 (2.6)	1.00
other complications	21 (21.9)	13 (13.5)	34 (17.7)	0.130

*TJA *Total joint arthroplasty, medical complications include cardiovascular, pulmonary, renal, thromboembolic (TVT), neurological and cerebrovascular AEs, delirium, urinary tract infection, electrolyte derangement and anemia requiring transfusion, surgical complications include periprosthetic joint infection (PJI), periprosthetic fracture (PP#), wound healing disorder (WHD) and hematoma, aseptic loosening (AL) and subsidence, dislocation, instability and wear of mobile components, range of motion limitation (ROM↓) and anterior knee pain (AKP), tendon rupture and nerve injuries, *Clavien-Dindo IV *life threatening complications leading to transfer to an intermediate care unit or intensive care unit, bold values = *p* ≤ 0.05; other complications include rare surgical and medical complications like bursitis trochanterica, unclear diarrhea, gastritris, blood sugar derailment, intra-operative soft tissue decollement

When analyzed separately for THA and TKA, revision-rates < 90 days for THA and TKA were no longer significant. Looking at the isolated total revision-rates in THA [31.6% (12) vs. 5.1% (2); *p* = 0.003] and TKA [25.9% (15) vs. 8.8% (5); *p* = 0.016] and the revision > 90 days in THA [21.1% (8) vs. 0% (0); *p* = 0.002] there are significant differences between the PD and the non-PD patients (see Appendix 1).

**Fig. 2:**
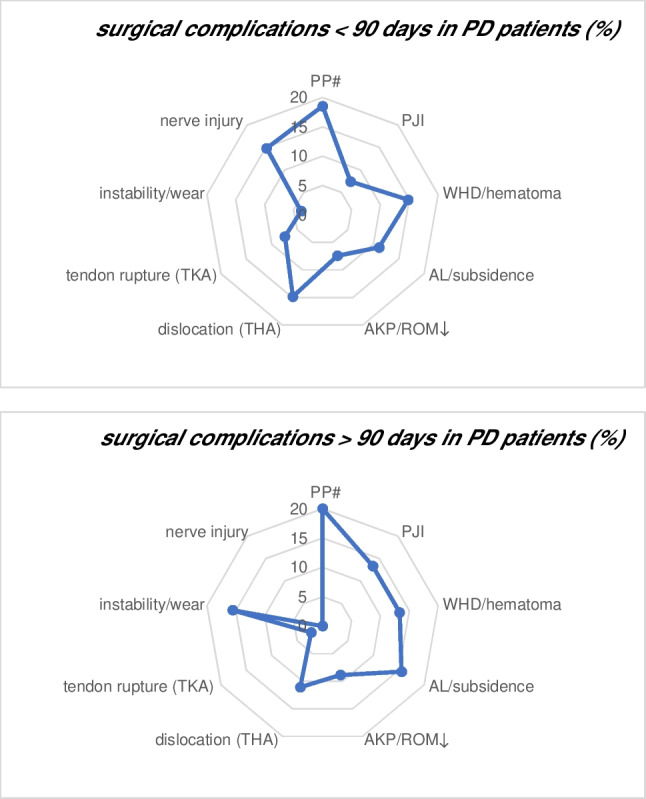
Percentage representation of the main reasons for revision-surgery < 90 days and > 90 days in PD patients. Surgical complications include periprosthetic joint infection (PJI), periprosthetic fracture (PP#), wound healing disorder (WHD) and hematoma, aseptic loosening (AL) and subsidence, dislocation, instability and wear of mobile components, range of motion limitation (ROM↓) and anterior knee pain (AKP), tendon rupture and nerve injuries

The main reasons for the revisions < 90 days (*n* = 16) included 19% (*n* = 5) periprosthetic fractures (TKA and THA), in each case 15% (*n* = 4) postoperative haematomas and wound healing disorders (TKA and THA), dislocation following acetabular cup malpositioning (THA) and nerve injuries. Otherwise, the main reasons for the revisions > 90 days were 20% (*n* = 9) periprosthetic fractures (TKA and THA), 16% (*n* = 7) aseptic loosening and subsidence and 16% (*n* = 7) instability or wear (TKA and THA). Detailed reasons for long-term revision-surgeries (< and > 90 days) are listed in Fig. 2.

### Primary outcome: postoperative complications

PD patients following TJA had significantly higher medical complications [68.8% (66) vs. 43.8% (42); *p* < 0.001] and higher surgical complications [40.6% (39) vs. 21.9% (21); *p* = 0.005] compared to the non-PD group (see Table 2 and Fig. 1). The medical complications subdivided into THA [73.7% (28) vs.51.3% (20); *p* = 0.043] and TKA [65.5% (38) vs. 38.6% (222); *p* = 0.004] still deliver significant outcomes. In contrast, the surgical complications show significant results only in the THA cases [44.7% (17) vs. 17.9% (7); *p* = 0.011] (see Appendix 1).

### Secondary outcome

An accurate breakdown of complications showed in particular higher rates of periprosthetic fracture, wound healing disorder and haematoma, aseptic loosening and subsidence, dislocation, instability and wear of mobile components, pulmonary AEs, electrolyte derangement, anemia requiring transfusion and delirium in the PD cohort (see Table 2).

PD patients had higher Hospital Frailty Risk Scores (HFRS) and ASA scores, reflecting their increased frailty and perioperative risk (see Table 1).

The PD population was also analyzed for fall events as a further outcome parameter. In the PD group, 20 out of 96 patients had recurrent falls, whereas in the non-PD group, only four out of 96 patients had recurrent falls (20,8% vs. 4,2%; *p* < 0.001).

There was no significant difference in revision-rates, surgical or medical complications between Parkinson's disease patients based on the type of joint replacement (hip or knee arthroplasty).

In total, there were five cases of postoperative transfers requiring intensive care according to the Clavien-Dindo-Classification Grade IV in our patient population. There were no significant differences between the groups (see Table 2).

### Multivariate logistic regression analysis

#### Primary outcome: revision rates

The multivariate logistic regression analysis identified Parkinson’s disease as a significant independent predictor of total revision-surgeries in TJA (OR 5.08, 95% CI 1.70–15.16, *p* = 0.004). Although no significant results could be obtained in the analyses of revisions < 90 days, Parkinsons disease was a significant predictor in revisions > 90 days (OR 10.1, 95% CI 1.94–52.93, *p* = 0.006).

Age also showed an independent effect on reoperation (OR 0.90, 95% CI 0.83–0.98, *p* = 0.019).

#### Primary outcome: postoperative complications

In Addition, PD was identified as a significant independent predictor of both surgical complications (OR 2.36, 95% CI 1.11–5.04, *p* = 0.026) and medical complications (OR 2.45, 95% CI 1.19–5.05, *p* = 0.015). Looking at medical complications in more detail, in addition to PD, type of joint replacement (TKA/THA) (OR 0.46, 95% CI 0.24–0.92, *p* = 0.027), age (OR 1.06, 95% CI 1.00–1.13, *p* = 0.043) and sex (OR 0.49, 95% CI 0.25–0.94, *p* = 0.031) were all independent predictive risk factors.

### Others

**Fig. 3:**
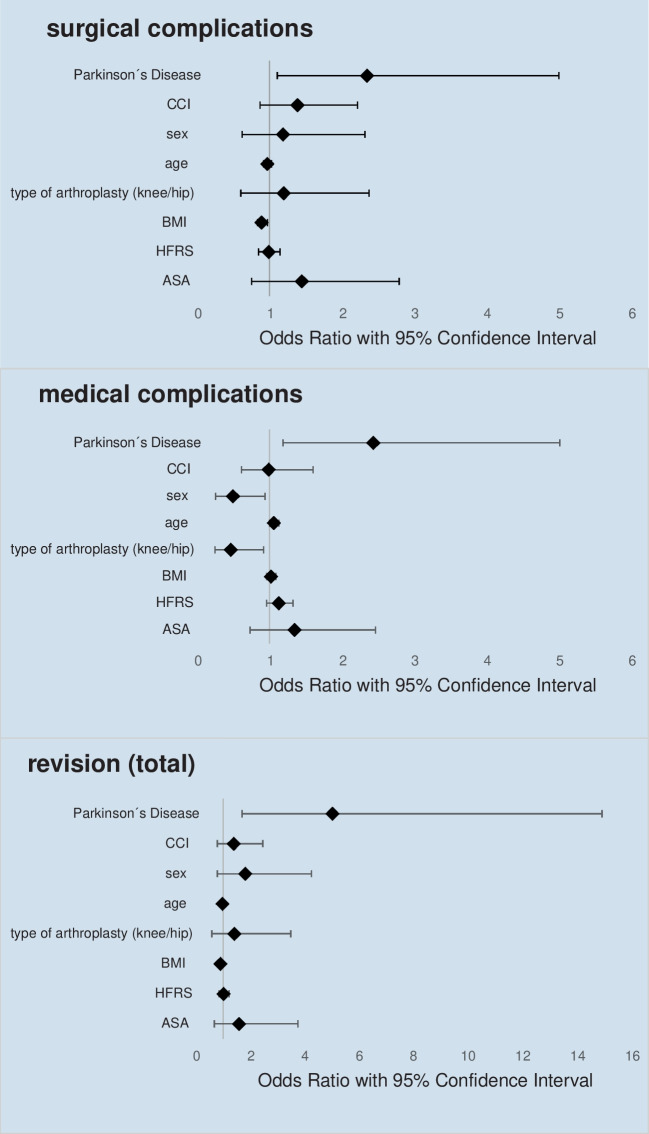
Results of the Multivariate logistic regression analysis in TJA. Forest-plots showing adjusted Odds Ratios and 95% Confidence interval. CCI = Charlson Comorbidity Index, BMI = Body Mass Index, HFRS = Hospital Frailty Risk Score, ASA = American Society of Anesthesiologists

Other variables included in the model to adjust for confounding factors, such as the HFRS, CCI and ASA score, were not significant predictive factors in any of the multivariate analyses. A graphical illustration of the multivariate logistic regression analysis **in TJA** can be found in the following forest-plots (see Fig. 3) and the detailed calculations for the multivariate analysis are given in Appendix 2.

## Discussion

### Baseline characteristics and group comparability

PD affects approximately 0.66% of patients in our cohort, with an average age of 71 years, aligning with reported prevalence rates of 1–2%, that rise up to 4% in older populations [23, 24]. This numbers are broadly in line with the literature, so this study shows a representative patient base. After the matching procedure the main baseline characteristics are not significantly different and support a good distribution of the patient clientele (see Table 1).

### Primary and secondary outcome:revison Surgeries and preoperative complications

PD was confirmed as a significant independent risk factor in multivariate logistic regression for long-term revision-surgeries (all TJAs) and revision-surgeries > 90 days, but not for the short-term revision-surgeries < 90 days. These findings can be explained with well-organized structures and preoperative patient preparation processes as a maximum care arthroplasty hospital. High-risk patients are efficiently filtered out preoperatively and referred to the internal medicine work-up, if preoperative risk factors are identified. This may explain the low risk of revision-surgeries < 90 days. However, this cannot be guaranteed in the long-term and outpatient setting (revision-surgeries > 90 days) due to the tendency of patients to fall and their frailty. According to Rondon et al. 23.6% of PD patients need a revision surgery at an average follow-up of 5.3 years, with periprosthetic infection, periprosthetic fracture and dislocation as main reasons for revision surgeries in TJA [25]. sons The main reasons for revisions < 90 days and > 90 days are listed in Fig. 2. These results are similar to those in the literature [25].

Some of the surgical complications required more than one surgical intervention. To understand all numbers in Table 2, it is also important to know, that some patients had revision-surgeries in both revision periods (< and > 90 days).

It is also notable that PD patients who had revision-surgeries > 90 days tended to have more subsequent revision surgeries (range: 1 – 12 revisions).

Looking at the pooled medical and surgical complications, PD was confirmed as an independent risk factor in both cases. A meta-analysis by Min et al. even indicates a 42% higher risk for medical complication rates (*p* = 0.004) of PD patients and a 65% higher risk for surgical complication rates (*p* = 0.010) compared to a matched cohort [14].

Our medical complications seem to be very high with 69% (PD) vs. 44% (non-PD). Anaemia and electrolyte imbalance were the most common medical complications. Appropriate geriatric co-management was only introduced during the study period, which contributed to more accurate assessment and treatment of electrolyte imbalances and appropriate blood management. The retrospective nature of this study may have introduced documentation bias, particularly regarding evolving clinical practices such as the use of cell savers in blood management and electrolyte correction protocols. Looking more closely at the medical complications, our results show an increased number of pulmonary adverse events and delirium in PD patients. Incidentally, it is known from other studies that PD patients are more likely to have pulmonary dysfunction and delirium compared to healthy subjects [26, 27].

A closer look at surgical complications also revealed results consistent with the current literature. In the area of endoprosthetic care, fractures, wound healing disorders and post-operative hematoma, aseptic loosening and stem subsidence, dislocation and instability, and abrasion were more common in PD patients [28]. Therefore, our study hypothesis was proportionate confirmed.

For the future it is important to optimize perioperative management and to implement prehabilitative assessments. This could be for example achieved via geriatric co-care, like clarification of internal diseases and especially neurological dopaminergic drug treatment, adjustment of metabolic disorders and osteoporosis, compensation of nutritional deficiencies, fall prevention strategies, delirium prophylaxis and preoperative walking support coaching.

### Further secondary outcome parameters

#### ASA and HFRS

As a secondary outcome point ASA score (2.7 vs. 2.3; *p* < 0.001) and HFRS score (4.6 vs. 2.4; *p* < 0.001) showed significantly higher values in the PD group. This finding shows, that PD patients are more “frail” and have a poorer preoperative condition. These scores were deliberately excluded from matching to investigate whether they independently influenced outcome. It is notable that ASA and “frailty” (HFRS) were identified as significant risk factors for the outcome parameters, but not as independent risk factors, although other research has already shown that there is a correlation between frailty and reduced outcome after TJA [29–31]. The influence of these two parameters will require further analysis in the future. Recent studies have shown, that joint replacement in combination with special orthogeriatric support is an effective therapy for improving “frailty” in hip and knee osteoarthritis patients [32]. Further scientific work-up is here useful to prevent PD related perioperative problems.

#### Fall events

As recurrent falls are increased in PD patients and are known to coincide with revisions and complications [15, 16], the fall events were investigated. In the PD group of the present study there was approximately a fivefold increased risk of falls compared to non-PD patients. In contrast to the standard literature [18], the number of falls of PD patients in the present study is with approximately 21% relatively low. This may be due to our highly specialized structure, which includes already fall prevention programmes and geriatric concepts.

#### BMI

As another secondary endpoint, BMI was a significant predictor for both revision-surgery and surgical complications, with higher BMI associated with lower odds of revision-surgery (OR 0.89, 95% CI 0.81–0.98, *p* = 0.013) and surgical complications (OR 0.90, 95% CI 0.83–0.97, *p* = 0.004). This relationship is not fully understood and could, for example, be related to a generally lower activity level in obese patients after joint replacement. According to Lübbeke et al., this could be associated with a lower number of mechanical revisions [33]. Another reason could be selection bias. A study by MacMahon et al. (2022) indicates that obese patients can achieve better postoperative results through careful preoperative assessment and preparation [34].

### Limitations

A limitation of the present study is the retrospective design, whereas errors in data collection cannot be ruled out with certainty, missing not collected parameters with a potential bias on the presented findings. Furthermore, the relatively small cohort of PD patients, while reflective of real-world prevalence, may limit the generalizability of the findings. Prospective, multicenter studies with larger sample sizes are needed to validate these results.

## Conclusion

Given the increased complication profile [35], patients with Parkinson's disease must be well medicated preoperatively and be carefully prepared for an artificial joint replacement as part of prehabilitation in order to avoid possible complications and reoperations. In particular, we recommend the application of a specific medical and orthogeriatric concept in these cases.

In conclusion, we do not categorize TJA as a contraindication for PD patients and consider TJA as a good treatment option in significantly improving the postoperative outcome of this patients.

At first sight the advantage of our analysis seems to be a relatively large study group with data analyzed over 14 years in a standardized treatment concept. Unfortunately, the final data set of 96 patients is quite small as a result of the low number of patients with PD. In percentage terms, this corresponds more or less to the data in the literature [23, 24]. With low postoperative complication rates in TJA, data analysis is nevertheless complex. An overall ten year survivorship of TJA for PD patients is estimated with only 72.3% [25]. It is therefore crucial to consider the disease related complications, revision-rates and the postoperative functional outcome. Further prospective randomized studies examining perioperative determinants, outcomes and long-term follow-up in an orthogeriatric setting would be useful to increase survivorship rates in the future.

## Data Availability

No datasets were generated or analysed during the current study.

## Appendix 1 Postoperative complications after elective total hip – or knee arthroplasty of patients with or without underlying Parkinson’s disease

**Table:**

		**Total Hip Arthroplasty**		
	**PD patients** *n* = 38 *n* (%)	**non-PD patients** *n* = 39 *n* (%)	**total** *n* = 77 *n *(%)	*p*-value
revision surgeries (number of patients)				
total revision	12 (31.6)	2 (5.1)	14 (18.2)	**0.003**
revision < 90 days	5 (13.2)	2 (5.1)	7 (9.1)	0.263
revision > 90 days	8 (21.1)	0 (0.0)	8 (10.4)	**0.002**
surgical complications (number of patients)	17 (44.7)	7 (17.9)	24 (31.2)	**0.011**
surgical complications < 90 days	7 (18.4)	5 (12.8)	12 (15.6)	0.498
surgical complications > 90 days	11 (28.9)	2 (5.1)	13 (16.9)	**0.005**
surgical complications (number of events)				
PP#	7 (18.4)	2 (5.1)	9 (11.7)	0.087
PJI	2 (5.3)	0 (0.0)	2 (2.6)	0.240
WHD/hematoma	4 (10.5)	0 (0.0)	4 (5.2)	0.055
AL/subsedience	6 (15.8)	0 (0.0)	6 (7.8)	**0.012**
AKP/ROM↓	1 (2.6)	0 (0.0)	1 (1.3)	0.494
dislocation (THA)	6 (15.8)	1 (2.6)	7 (9.1)	0.056
tendon rupture (TKA)	0	0	0	**-**
instability/wear	4 (10.5)	0 (0.0)	4 (5.2)	0.055
nerval injury	0	0	0	-
medical complications (number of patients)	28 (73.7)	20 (51.3)	48 (62.3)	**0.043**
medical complications < 90 days	24 (63.2)	20 (51.3)	44 (57.1)	0.292
medical complications > 90 days	5 (13.2)	0 (0.0)	5 (6.5)	**0.025**
medical complications (number of events)				
pulmonary AEs	5 (13.2)	0 (0.0)	5 (6.5)	**0.025**
decubitus	2 (5.3)	2 (5.1)	4 (5.2)	1.00
cardiovascular AEs	1 (2.6)	1 (2.6)	2 (2.6)	1.00
renal AEs	2 (5.3)	3 (7.7)	5 (6.5)	1.00
electrolyte derangement	12 (31.6)	6 (15.4)	18 (23.4)	0.093
anemia	19 (50.0)	15 (38.5)	34 (44.2)	0.308
delirium	10 (26.3)	1 (2.6)	11 (14.3)	**0.003**
urinary tract infection	1 (2.6)	0 (0.0)	1 (1.3)	0.494
TVT	1 (2.6)	0 (0.0)	1 (1.3)	0.494
other complications (number of events)				
Clavien-Dindo IV	1 (2.6)	1 (2.6)	2 (2.6)	1.00
other complications	13 (34.2)	7 (17.9)	20 (2.0)	0.125
		**Total Knee Arthroplasty**		
	**PD patients** *n* = 58 *n* (%)	**non-PD patients** *n* = 57 *n* (%)	**total** *n* = 115 *n* (%)	*p*-value
revision surgeries (number of patients)				
total revision	15 (25.9)	5 (8.8)	20 (17.4)	**0.016**
revision < 90 days	8 (13.8)	3 (5.3)	11 (9.6)	0.120
revision > 90 days	8 (13.8)	2 (3.5)	10 (8.7)	0.094
surgical complications (number of patients)	22 (37.9)	14 (24.6)	36 (31.3)	0.122
surgical complications < 90 days	14 (24.1)	8 (14.0)	22 (19.1)	0.168
surgical complications > 90 days	11 (19.0)	8 (14.0)	19 (16.5)	0.477
surgical complications (number of events)				
PP#	7 (12.1)	1 (1.8)	8 (7.0)	0.061
PJI	6 (10.3)	2 (3.5)	8 (7.0)	0.272
WHD/hematoma	6 (10.3)	3 (5.3)	9 (7.8)	0.490
AL/subsedience	4 (6.9)	0 (0.0)	4 (3.5)	0.119
AKP/ROM↓	6 (10.3)	8 (14.0)	14 (12.2)	0.545
dislocation (THA)	3 (5.2)	0 (0.0)	3 (2.6)	0.243
tendon rupture (TKA)	3 (5.2)	0 (0.0)	3 (2.6)	0.243
instability/wear	4 (6.9)	0 (0.0)	4 (3.5)	0.119
nerval injury	4 (6.9)	3 (5.3)	7 (6.1)	1.00
medical complications (number of patients)	38 (65.5)	22 (38.6)	60 (52.2)	**0.004**
medical complications < 90 days	34 (58.6)	22 (38.6)	56 (48.7)	**0.032**
medical complications < 90 days	4 (6.9)	0 (0.0)	4 (3.5)	0.119
medical complications (number of events)				
pulmonary AEs	5 (8.6)	1 (1.8)	6 (5.2)	0.206
decubitus	0	0	0	-
nerval injury	4 (6.9)	3 (5.3)	7 (6.1)	1.00
cardiovascular AEs	6 (10.3)	1 (1.8)	7 (6.1)	0.114
renal AEs	4 (6.9)	3 (5.3)	7 (6.1)	1.00
electrolyte derangement	16 (27.6)	5 (8.8)	21 (18.3)	**0.009**
anemia	24 (41.4)	14 (24.6)	38 (33.0)	0.055
delirium	4 (6.9)	3 (5.3)	7 (6.1)	1.00
urinary tract infection	3 (5.2)	2 (3.5)	5 (4.3)	1.00
TVT	3 (5.2)	0 (0.0)	3 (2.6)	0.243
other complications (number of events)				
Clavien-Dindo IV	1 (1.7)	2 (3.5)	3 (2.6)	0.618
other complications	8 (13.8)	6 (10.5)	14 (12.2)	0.592

* THA *Total hip arthroplasty, TKA = Total Knee arthroplasty, medical complications include cardiovascular, pulmonary, renal, thromboembolic (TVT), neurological and cerebrovascular AEs, delirium, urinary tract infection, electrolyte derangement and anemia requiring transfusion, surgical complications include periprosthetic joint infection (PJI), periprosthetic fracture (PP#), wound healing disorder (WHD) and hematoma, aseptic loosening (AL) and subsdence, dislocation, instability and wear of mobile components, range of motion limitation (ROM↓) and anterior knee pain (AKP), tendon rupture and nerve injuries, *Clavien-Dindo IV *life threatening complications leading to transfer to an intermediate care unit or intensive care unit, bold values = *p* ≤ 0.05; other complications include rare surgical and medical complications like bursitis trochanterica, unclear diarrhea, gastritris, blood sugar derailment, intra-operative soft tissue decollement

## Appendix 2: Results of the multivariate logistic regression analysis

**Table:**

Revision (total):								
	B	SE	Wald	df	p-value	Odds Ratio	95% Confidence Intervall	
							Lower	Upper
Parkinson’s Disease (1)	1,625	,558	8,471	1	**,004**	5,077	1,700	15,161
HFRS	,012	,096	,016	1	,899	1,012	,839	1,221
BMI	-,121	,049	6,112	1	**,013**	,886	,805	,975
Type of arthroplasty	,348	,466	,557	1	,455	1,416	,568	3,529
Age	-,028	,035	,627	1	,429	,973	,908	1,042
Sex	,597	,438	1,855	1	,173	1,816	,770	4,287
CCI	,329	,295	1,244	1	,265	1,389	,780	2,475
ASA	,468	,442	1,123	1	,289	1,597	,672	3,797
Konstante	,021	2,632	,000	1	,994	1,021		

Variables included: Parkinson’s Disease, *CCI *Charlson Comorbidity Index, *BMI *Body Mass Index, *HFRS *Hospital Frailty Risk Score, *ASA *American Society of Anesthesiologists, Type of arthroplasty (knee/hip), Age, Sex, bold values = *p* ≤ 0.05

**Table:**

Revision < 90 days:								
	B	SE	Wald	df	p-value	Odds Ratio	95% Confidence Intervall	
							Lower	Upper
Parkinson’s Disease (1)	1,025	,680	2,275	1	,131	2,787	,736	10,557
HFRS	-,011	,122	,008	1	,928	,989	,779	1,255
BMI	-,099	,062	2,512	1	,113	,906	,802	1,024
Type of arthroplasty	,084	,572	,022	1	,883	1,088	,354	3,341
Age	,071	,049	2,097	1	,148	1,073	,975	1,181
Sex	,101	,535	,036	1	,851	1,106	,387	3,159
CCI	,121	,352	,118	1	,732	1,129	,566	2,252
ASA	,279	,566	,243	1	,622	1,322	,436	4,013
Konstante	-6,462	3,864	2,797	1	,094	,002		

Variables included: Parkinson’s Disease, *CCI *Charlson Comorbidity Index, *BMI *Body Mass Index, *HFRS *Hospital Frailty Risk Score, *ASA *American Society of Anesthesiologists, Type of arthroplasty (knee/hip), Age, Sex, bold values = *p* ≤ 0.05

**Table:**

Revision > 90 days:								
	B	SE	Wald	df	p-value	Odds Ratio	95% Confidence Intervall	
							Lower	Upper
Parkinson’s Disease (1)	2,316	,844	7,535	1	**,006**	10,131	1,939	52,934
HFRS	-,061	,129	,223	1	,637	,941	,730	1,212
BMI	-,089	,063	1,987	1	,159	,915	,808	1,036
Type of arthroplasty	,293	,585	,251	1	,616	1,341	,426	4,223
Age	-,102	,043	5,484	1	**,019**	,903	,829	,984
Sex	1,054	,589	3,204	1	,073	2,869	,905	9,098
CCI	,480	,375	1,637	1	,201	1,616	,775	3,371
ASA	,238	,563	,179	1	,673	1,269	,421	3,824
Konstante	3,158	3,141	1,011	1	,315	23,524		

Variables included: Parkinson’s Disease, *CCI *Charlson Comorbidity Index, *BMI *Body Mass Index, *HFRS *Hospital Frailty Risk Score, *ASA *American Society of Anesthesiologists, Type of arthroplasty (knee/hip), Age, Sex, bold values = *p* ≤ 0.05

**Table:**

Surgical complications:								
	B	SE	Wald	df	p-value	Odds Ratio	95% Confidence Intervall	
							Lower	Upper
Parkinson’s Disease (1)	,860	,386	4,964	1	**,026**	2,363	1,109	5,037
HFRS	-,012	,078	,024	1	,877	,988	,849	1,151
BMI	-,108	,037	8,342	1	**,004**	,897	,834	,966
Type of arthroplasty	,183	,352	,269	1	,604	1,201	,602	2,394
Age	-,028	,029	,898	1	,343	,973	,918	1,030
Sex	,180	,339	,280	1	,596	1,197	,615	2,328
CCI	,333	,239	1,944	1	,163	1,396	,874	2,230
ASA	,374	,336	1,237	1	,266	1,454	,752	2,810
Konstante	1,747	2,142	,665	1	,415	5,738		

Variables included: Parkinson’s Disease, *CCI* Charlson Comorbidity Index, *BMI* Body Mass Index, *HFRS* Hospital Frailty Risk Score, *ASA* American Society of Anesthesiologists, Type of arthroplasty (knee/hip), Age, Sex, bold values = *p* ≤ 0.05

**Table:**

Medical Complications:								
	B	SE	Wald	df	p-value	Odds Ratio	95% Confidence Intervall	
							Lower	Upper
Parkinson’s Disease (1)	,897	,369	5,905	1	**,015**	2,452	1,189	5,054
HFRS	,118	,083	2,020	1	,155	1,126	,956	1,325
BMI	,021	,034	,378	1	,539	1,021	,955	1,091
Type of arthroplasty	-,767	,348	4,859	1	**,027**	,464	,235	,918
Age	,061	,030	4,088	1	**,043**	1,063	1,002	1,127
Sex	-,720	,333	4,657	1	**,031**	,487	,253	,936
CCI	-,007	,247	,001	1	,977	,993	,612	1,611
ASA	,297	,313	,898	1	,343	1,345	,729	2,484
Konstante	-5,403	2,152	6,302	1	**,012**	,005		

Variables included: Parkinson’s Disease, *CCI *Charlson Comorbidity Index, *BMI *Body Mass Index, *HFRS *Hospital Frailty Risk Score, *ASA *American Society of Anesthesiologists, Type of arthroplasty (knee/hip), Age, Sex, bold values = *p* ≤ 0.05
